# FANCD2 regulates BLM complex functions independently of FANCI to promote replication fork recovery

**DOI:** 10.1093/nar/gkt348

**Published:** 2013-05-08

**Authors:** Indrajit Chaudhury, Archana Sareen, Maya Raghunandan, Alexandra Sobeck

**Affiliations:** Department of Biochemistry, Molecular Biology and Biophysics, University of Minnesota, Minneapolis, MN 55455, USA

## Abstract

Fanconi Anemia (FA) and Bloom Syndrome share overlapping phenotypes including spontaneous chromosomal abnormalities and increased cancer predisposition. The FA protein pathway comprises an upstream core complex that mediates recruitment of two central players, FANCD2 and FANCI, to sites of stalled replication forks. Successful fork recovery depends on the Bloom’s helicase BLM that participates in a larger protein complex (‘BLMcx’) containing topoisomerase III alpha, RMI1, RMI2 and replication protein A. We show that FANCD2 is an essential regulator of BLMcx functions: it maintains BLM protein stability and is crucial for complete BLMcx assembly; moreover, it recruits BLMcx to replicating chromatin during normal S-phase and mediates phosphorylation of BLMcx members in response to DNA damage. During replication stress, FANCD2 and BLM cooperate to promote restart of stalled replication forks while suppressing firing of new replication origins. In contrast, FANCI is dispensable for FANCD2-dependent BLMcx regulation, demonstrating functional separation of FANCD2 from FANCI.

## INTRODUCTION

Fanconi anemia (FA) and Bloom syndrome (BS) are genomic instability diseases that predispose affected individuals to cancer. FA is characterized by bone marrow failure, congenital abnormalities and a high risk to develop leukemia and squamous cell carcinomas. FA cells are sensitive to DNA interstrand crosslinks (ICLs) and show spontaneous chromosomal aberrations that are further exacerbated on treatment with replication-inhibiting agents (1,2). Fifteen known FA proteins act in a common pathway that is activated when the replication machinery encounters DNA damage. On replication fork stalling, the upstream FA core complex (composed of eight FA proteins) is recruited to chromatin by one of its members, FANCM (3–5). The core complex then monoubiquitinates the central FA pathway proteins FANCD2 and FANCI that subsequently localize to chromatin and into DNA repair foci (6,7). Monoubiquitinated FANCD2 (FANCD2^Ub^) functions to recruit DNA repair factors FAN1 (Fanconi-associated nuclease 1) (8–11) and SLX4 (identical to FANCP; a Holliday junction (HJ) resolvase in complex with SLX1) (12–15), suggesting that chromatin-bound FANCD2^Ub^ is a docking platform for certain DNA repair nucleases. Positioned downstream in the FA pathway are the breast cancer–associated proteins FANCD1/BRCA2 (breast cancer–associated protein 2), FANCN/PALB2 (partner and localizer of BRCA2) and FANCJ (BRIP1, BRCA1-interacting protein 1) that function in homologous recombination (HR) repair of DNA double-stranded breaks (DNA DSBs) (16,17). Intriguingly, recent studies identified a DSB repair-independent function of BRCA2—in concert with FANCD2—to protect stalled replication forks from degradation by the MRE11 nuclease (2,18).

BS is closely related to FA, characterized by growth abnormalities, immunodeficiency and an increased risk to develop hematological and solid tumors. BS and FA cells share phenotypical features including DNA ICL sensitivity and spontaneous chromosomal aberrations (19,20). The single BS protein, BLM, is a RecQ helicase that participates in a protein complex (‘BLMcx’) containing topoisomerase III alpha (TOP3a), RMI1, RMI2 and the replication protein A heterotrimer (RPA1-3) (21–24). BLMcx promotes dissolution of HJs—mobile DNA crossover structures that arise during HR-mediated repair of DNA DSBs (25–27). Intriguingly, HJ structures also form during replication fork recovery (28,29), and it was recently shown that BLM and RMI1 mediate the restart of stalled replication forks *in vivo* (30,31).

Accumulating evidence suggests functional interactions between the FA and BLM pathways: (i) The upstream FA core complex and BLMcx can form a larger complex using FANCM as linker protein (3,24); moreover, the FA core complex mediates DNA ICL-induced recruitment of BLM and RPA to DNA and into DNA repair foci (3,32–34). (ii) The downstream FA pathway protein FANCJ protects BLM protein stability and cooperates with BLM to unwind damaged DNA duplex substrates (35). (iii) The central FA pathway protein FANCD2 co-immunoprecipitates with BLM from ICL-treated human cells (32,33); moreover, BLM and TOP3a are epistatic to FANCD2 to mediate cellular DNA ICL resistance (20).

Importantly, FANCD2 and BLM also prevent replication fork collapse during unperturbed S-phase (36,37), indicating that these proteins communicate in the context of fork stalling. However, if and how FANCD2 acts in concert with BLM and other BLMcx members to mediate replication fork recovery, and whether the FANCD2 dimerization partner FANCI is involved in these processes, is not known.

We combined *Xenopus laevis* egg extracts and human cell-based assays to investigate a putative functional connection between FANCD2, FANCI and BLMcx. Our results indicate that FANCD2 is an integral stabilizing member of BLMcx that recruits the entire complex to replicating chromatin and controls DNA damage-triggered phosphorylation of BLMcx members. Following replication fork stalling, FANCD2 and BLM cooperate to promote fork restart. Strikingly, FANCI is not required for FANCD2-dependent BLMcx regulation, supporting our recent finding that FANCD2 ‘dissociates’ from FANCI on FA pathway activation (38) and demonstrating a separation of function between FANCD2 and FANCI.

## MATERIALS AND METHODS

### Preparation of Xenopus egg extracts

S-phase extracts were prepared from *Xenopus* eggs as described (36,39). Where indicated, extracts were treated with 100 µM MG132.

### Preparation of dsDNA substrates

Circular plasmid DNA (pBSKS) was linearized by digestion with EcoRV and used at a concentration of 50 ng/µl in egg extracts.

### Chromosomal replication assay in Xenopus egg extracts

S-phase extracts were supplemented with 1000 *X.laevis* sperm nuclei/µl. Reaction aliquots were pulse labeled with [α-^32^P]dGTP at the indicated time windows. Reactions were stopped with 1% SDS/40 mM EDTA (pH 7.8) and digested with proteinase K (1 mg/ml) at 37°C for 1 h. DNA was extracted with phenol-chloroform and electrophoresed on an agarose gel. Gels were dried and exposed to X-ray film.

### Preparation of chromatin fractions from Xenopus egg extracts

At indicated time points, 50 µl of S-phase egg extracts containing 1000 sperm nuclei/µl were diluted in chromatin isolation buffer (40 mM HEPES, 100 mM KCl, 20 mM MgCl_2_, 0.2% Triton X-100) and purified by centrifugation through a 30% (wt/vol) sucrose cushion for 25 min at 6000*g* at 4°C. Chromatin pellets were analyzed by gel electrophoresis and immunoblotting.

### Immunofluorescence analysis in Xenopus nuclei

Replicating nuclei were re-isolated from *Xenopus* egg extracts following a protocol from the Heald laboratory (40). Briefly, nuclei were re-isolated from extract/nuclei mixes via centrifugation through a 40% glycerol cushion onto coverslips. Nuclei were fixed onto coverslips with 3.7% formaldehyde, permeabilized with PBS containing 0.2% Triton X-100 and blocked with 7.5% BSA. Primary and secondary antibodies were diluted in PBS containing 0.5% BSA. Nuclei were incubated in primary antibody [1:1000 for anti-BLM (rabbit); 1:1000 for anti-RPA2 (rat); 1:3000 for anti-RPA2^S33-P^ (rabbit) and 1:2000 for anti-FANCD2 (rabbit)] for 1 h at 4°C. Following five 5-min washes with PBS, secondary antibody was added at 1:1000 (Alexa Fluor 594-conjugated goat anti-rabbit or Alexa Fluor 488-conjugated goat anti-rabbit; Molecular Probes). Nuclei were stained with Hoechst 33342 dye (Molecular Probes) and then mounted with anti-fade mounting solution (Vectashield, Vector Laboratories). For analysis of nuclear foci formation, wide-field images were captured using the Axio Imager A1 (Zeiss). Images were processed using Image J software (NIH). Single nuclei were scored for BLM foci and RPA2^S33-P^ foci. Evaluation of foci-containing nuclei was performed using Microsoft Excel.

### Immunodepletion of Xenopus egg extracts

Immunodepletions were performed essentially as previously described (36). In brief, 200 µl of Sepharose 4B beads (50% slurry) were coupled to 100 µl of protein-specific antibody (FANCD2 or FANCI) or corresponding IgG control serum. Beads were pelleted and washed in XB-buffer. For depletion, 200 µl of extract was added to 100 µl of dry conjugated beads and incubated on ice for 60 min. The extract-bead mix was centrifuged at 1800*g* for 5 min, and the extract was separated from the bead pellet. For quantitative protein removal, one to two depletion rounds were performed.

### Immunoprecipitation from Xenopus egg extracts

Hundred microliters of Sepharose 4B beads (50% slurry) were coupled to 10–20 µl of affinity-purified protein-specific BLM antibody or control (IgG) antibody. Eighty microliters of extracts were diluted in 1200 µl of immunoprecipitation (IP) buffer (10 mM Tris, pH 7.4, 150 mM NaCl, 1% NP-40, 0.5% Sodium Deoxycholate, 1 mM EDTA, 1 mM DTT, 0.5 mg/ml pefabloc protease inhibitor) and centrifuged at 40 000*g* for 20 min. Antibody-coupled beads were added to the supernatant and the mix was rotated overnight at 4°C. IP beads were washed in IP buffer, boiled in 1× NuPAGE loading buffer (Invitrogen) and analyzed for bound proteins by SDS-PAGE and western blotting.

### Immunoblotting

Protein samples were separated on gradient gels and transferred to Immobilon P membranes (Millipore). After blocking in 5% milk, membranes were incubated with the following primary antibodies. *Xenopus* egg extracts: FANCD2 (1:2000), FANCI (1:1000), BLM (1:1000), RMI1 (1:250), RPA1-3 (1:2000), histone H3 (1:6000) or Myc (1:1000). Human cells: FANCD2 (1:1000), FANCI (1:1000), BLM (1:1500), TOP3A (1:1000), RPA1 (1:1000), histone H3 (1:6000), CDA (1:500) and GAPDH (1:1000). Horseradish peroxidase-conjugated rabbit secondary antibody (Jackson Labs) or mouse secondary antibody (Biorad) was used at dilutions of 1:10 000 and 1:4000, respectively. Protein bands were visualized using an ECL Plus system (Amersham).

### Protein purification

*Xenopus laevis* recombinant myc-FANCD2_WT_ and myc-FANCD2_K562R_ proteins were purified as described (38). Purified proteins are shown in Supplementary Figure S1.

### Antibodies

*Xenopus laevis*. Antibodies against FANCD2 and FANCI were previously described (38). Polyclonal rabbit antibodies were raised against the N- and C-termini of BLM (MAALPQNNLQKQLELFPAKG and MAPPMPQPNRRFLKPSYSMF). Antibodies against TOP3a were a gift from W. Dunphy, and antibodies against RPA subunits 1–3 were a gift from K. Cimprich. *Homo sapiens*. Commercial antibodies were used against human RMI1 (GTX11686; crossreactive with *Xenopus* RMI1), Myc (Sigma, C3956; Covance, 14865101), FANCD2 (abcam, ab2127), FANCI (Bethyl, A300-213A), BLM (abcam, ab476), TOP3a (Proteintech, 14525-1-AP), RPA1 (Calbiochem, NA-13), CDA (abcam, ab56053), histone H3 (abcam, ab1791), GAPDH (Genetex, GTX627408), total RPA2 (Cell Signaling, 2208) and phospho-RPA2 (phospho-S33; Bethyl, A300-246) (both RPA2 antibodies are crossreactive with *X.laevis* RPA2).

### Preparation of whole cell extracts and cell fractions from human cells

For whole cell extract (WCE) preparation, cells were washed in PBS, resuspended in lysis buffer (10 mM Tris, pH 7.4, 150 mM NaCl, 1% NP-40, 0.5% Sodium Deoxycholate, 1 mM EDTA, 1 mM DTT, 0.5 mg/ml pefabloc protease inhibitor) and incubated on ice for 20 min. Cell extracts were centrifuged for 5 min at 10 000 *g*, and the supernatant was used for further analysis. Cytoplasmic and chromatin fractions were prepared using the Subcellular Protein Fractionation Kit (Thermo Scientific).

### IP from human cells>

Untreated or aphidicolin-treated PD20 or PD20 + D2 cells were lysed in buffer containing 10 mM Tris, pH 7.4, 150 mM NaCl, 1% NP-40, 0.5% Sodium Deoxycholate, 1 mM EDTA, 1 mM DTT, 0.5 mg/ml protease inhibitor (Boeringer). Lysates were precleared with rabbit IgG and subjected to IP with FANCD2, BLM or IgG antibody at 4°C overnight. Hundred microliter of Sepharose 4B beads (50% slurry) were added and rotated for 30 min at 4°C. Beads were pelleted from solution, washed in cell lysis buffer, boiled in 1× NuPAGE buffer (Invitrogen) and analyzed for the presence of proteins by SDS-PAGE and western blotting.

### siRNA experiments

siRNA duplexes were purchased from Dharmacon research (Thermo Scientific, USA). The sequence of FANCD2 siRNA is CAACAUACCUCGACUCAUUUU (10). For BLM, siGENOME SMARTpool siRNA consisting of four siRNA duplexes with target sequences GAGCACAUCUGUAAAUUAA, GAGAAACUCACUUCAAUAA, CAGGAUGGCUGUCAGGUUA and CUAAAUCUGUGGAGGGUUA was used. siGENOME non-targeting siRNA was used as a control. Transfections were performed using DharmaFECT1 transfection reagent according to the manufacturer’s protocol.

### DNA fiber assay

We used a previously described DNA fiber protocol (41). Moving replication forks were labeled with digoxigenin-dUTPs (DigU) for 25 min and then with biotin-dUTPs (BioU) for 40 min. To allow efficient incorporation of the dUTPs, a hypotonic buffer treatment (10 mM HEPES, 30 mM KCl, pH 7.4) preceded each dUTP-labeling step. To visualize labeled fibers, cells were mixed with a 10-fold excess of unlabeled cells, fixed and dropped onto slides. After cell lysis, DNA fibers were released and extended by tilting the slides. Incorporated dUTPs were visualized by immunofluorescence detection using anti-digoxigenin-Rhodamine (Roche) and streptavidin-Alexa-Fluor-488 (Invitrogen). Images were captured using a Deltavision microscope (Applied Precision) and analyzed using Deltavision softWoRx 5.5 software. All shown DNA fiber results are means of three independent experiments (300 DNA fibers/experiment). Statistics were calculated using Prism software (Supplementary Table S1). Error bars show s.e.m. *P* values were determined using Mann–Whitney test.

## RESULTS

### FANCD2 mediates chromatin recruitment of BLMcx independently of FANCI

**Figure 1.**
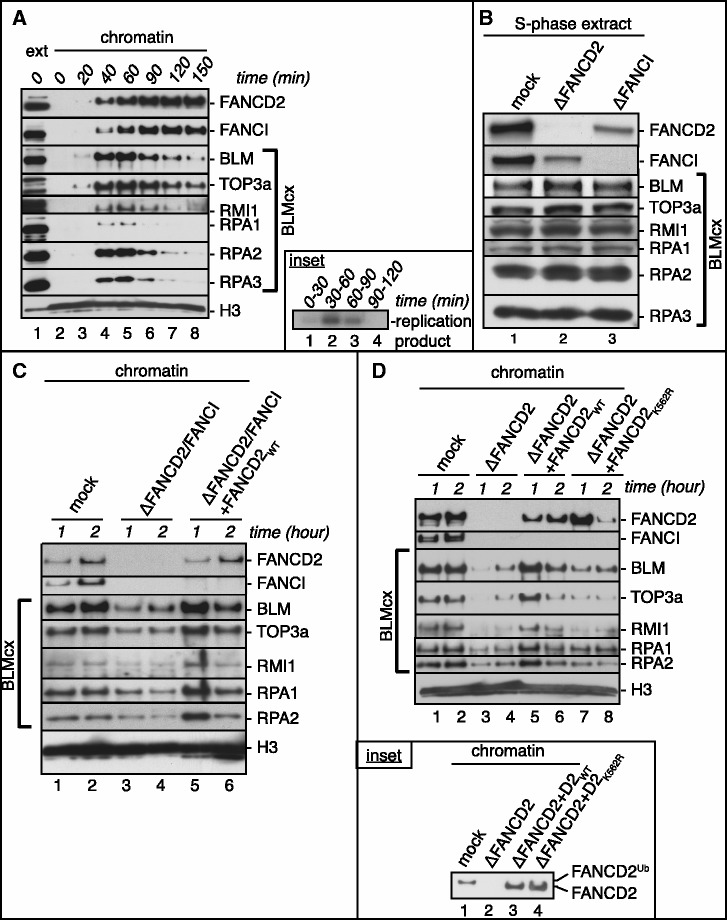
FANCD2 recruits BLMcx to replicating chromatin independently of FANCI. (**A**) FANCD2, FANCI and BLMcx members exhibit overlapping chromatin-binding patterns in S-phase. Sperm chromatin was replicated in *Xenopus* S-phase extracts and re-isolated at the indicated time points. Chromatin fractions (lanes 2–8) were analyzed for bound FANCD2, FANCI and BLMcx members. Lane 1: 1 µl extract (loading control). Inset: replication assay. Replication was monitored by pulsing replicating extract aliquots with [α-^32^P]dGTP at the indicated time windows. (**B**) Immunodepletion of FANCD2 or FANCI from S-phase extracts does not co-deplete BLMcx proteins. S-phase extracts were mock-, FANCD2 or FANCI-depleted and analyzed for the presence of FANCD2, FANCI and BLMcx members. (**C**) Recombinant FANCD2_WT_ restores BLMcx recruitment to replicating chromatin in FANCD2/FANCI double-depleted extracts. S-phase extracts were mock depleted (lanes 1 and 2), FANCD2/FANCI depleted (lanes 3 and 4) or FANCD2/FANCI depleted and reconstituted with myc–FANCD2_WT_ (lanes 5 and 6). Sperm chromatin was allowed to replicate in the different extracts, isolated at the indicated time points and analyzed for bound FANCD2, FANCI and BLMcx members. (**D**) Recombinant FANCD2_WT_—but not FANCD2_K562R_—restores BLMcx recruitment to replicating chromatin in FANCD2-depleted extracts. S-phase extracts were mock depleted (lanes 1 and 2), FANCD2 depleted (lanes 3 and 4) or FANCD2 depleted and reconstituted with either myc–FANCD2_WT_ (lanes 5 and 6) or myc-FANCD2_K562R_ (lanes 7 and 8). Sperm chromatin was replicated in the different extracts, re-isolated at indicated time points and analyzed for bound FANCD2, FANCI and BLMcx members. Inset: FANCD2-depleted extracts fail to promote monoubiquitination of supplemented myc-FANCD2_WT_ (owing to low residual FANCI levels) but support chromatin recruitment of non-ubiquitinated myc-FANCD2_WT_ and myc-FANCD2_K562R_. Chromatin fractions isolated from mock-depleted (lane 1) or FANCD2-depleted (lane 2) extracts, or from FANCD2-depleted extracts supplemented with myc-FANCD2_WT_ (lane 3) or myc-FANCD2_K562R_ (lane 4) were run on a low-percentage gel to distinguish non-ubiquitinated from monoubiquitinated FANCD2 isoforms.

Using naturally synchronous *Xenopus* S-phase extracts, we and others previously showed that FA and BLM pathway proteins bind chromatin in a replication-dependent manner (36,37,42). Comparing chromatin-binding behavior of FANCD2, FANCI and BLMcx members BLM, RMI1, TOP3a and RPA1-3 revealed that these proteins began to associate with chromatin in early S-phase and remained bound throughout replication (Figure 1A). BLMcx members began to dissociate from chromatin once replication was completed, whereas FANCD2 and FANCI remained chromatin-bound as previously described (36,38), hinting that FANCD2 and FANCI have additional post-replicative functions. Next, we asked if chromatin recruitment of BLMcx depended on FANCD2 or FANCI. Because BLM co-immunoprecipitates FANCD2 from human cells (32,33), we initially tested if depletion of FANCD2 or FANCI co-depleted BLMcx members from egg extracts. As expected, depletion of FANCD2 co-depleted ∼80% of FANCI and *vice versa* (38,42). In contrast, protein levels of BLMcx members were unaffected in FANCD2 or FANCI-depleted extracts (Figure 1B), demonstrating that the majority of FANCD2 and FANCI molecules do not interact with BLMcx members in DNA-free extracts. To first test if BLMcx chromatin binding depended on FANCD2 or FANCI, chromatin was allowed to replicate in mock- or FANCD2/FANCI double-depleted extracts and re-isolated at different time points. Chromatin recruitment of all BLMcx members was substantially reduced in FANCD2/FANCI double-depleted extracts (Figure 1C). To determine which FA protein was responsible for BLMcx chromatin recruitment, we took advantage of our recent finding that recombinant wild-type FANCD2 (myc-FANCD2_WT_) binds replicating chromatin in absence of FANCI, whereas the reverse is not the case (38). Addition of myc-FANCD2_WT_ to FANCD2/FANCI double-depleted extracts restored chromatin binding of all BLMcx members (Figure 1C, lanes 5 and 6), indicating that FANCD2 *alone* is sufficient to recruit BLMcx to replicating chromatin. In further support, FANCD2-depleted extracts (containing 20% residual FANCI) failed to recruit BLMcx to chromatin (Figure 1D), but were rescued by adding myc-FANCD2_WT_. Moreover, FANCI-depleted extracts (containing 20% residual FANCD2 that still partially associates with chromatin) showed a much less pronounced reduction in chromatin-bound BLMcx levels that was again rescued by adding myc-FANCD2_WT_ (Supplementary Figure S2). Thus, FANCD2 mediates chromatin recruitment of BLMcx independently of FANCI.

In human cells, BLM and a RPA2 isoform phosphorylated at serine 33 (RPA2^S33-P^) colocalize in late S-phase nuclear foci, likely at unresolved replication intermediates (43). We tested if FANCD2 was responsible for recruiting BLM and RPA2^S33-P^ to late S-phase nuclear foci. Like their human counterparts, *Xenopus* BLM and RPA2 colocalized in chromatin foci in replicating nuclei, particularly during late replication; moreover, FANCD2 colocalized with RPA2 in late S-phase foci as well (Supplementary Figure S3A). BLM and RPA2^S33-P^ foci formation was inhibited in FANCD2-depleted extracts and restored by adding myc-FANCD2_WT_ (Supplementary Figure S3B–D). Thus, FANCD2 recruits BLM and RPA2^S33-P^ to late S-phase foci, possibly at sites of unresolved replication structures.

### FANCD2 monoubiquitination is dispensable for BLMcx chromatin recruitment

FANCI-depleted extracts support chromatin recruitment of residual endogenous FANCD2, as well as recombinant myc-FANCD2_WT_, although FANCD2 is not monoubiquitinated in absence of FANCI (38). Importantly, myc-FANCD2_WT_ added to FANCD2-depleted extracts is not monoubiquitinated either, likely because the residual FANCI levels (20%) in these extracts are not sufficient to promote monoubiquitination of myc-FANCD2_WT_ (Figure 1D, inset). Thus, the fact that myc-FANCD2_WT_ restored BLMcx chromatin binding in FANCD2 and FANCD2/FANCI double-depleted extracts (see Figure 1C and D) indicated that FANCD2^Ub^ formation is dispensable for BLMcx recruitment to chromatin and into nuclear foci. For further investigation, we compared chromatin recruitment and foci formation of BLMcx members in FANCD2-depleted extracts reconstituted with myc-FANCD2_WT_ or with a monoubiquitination-dead FANCD2 mutant (myc-FANCD2_K562R_). Interestingly, unlike chromatin-bound ‘non-ubiquitinated’ FANCD2_WT_, the chromatin-bound myc-FANCD2_K562R_ mutant did not fully restore chromatin recruitment of BLMcx (Figure 1D), or foci formation of BLM and RPA2^S33-P^ (Supplementary Figure S3B–D). We also noticed that myc-FANCD2_K562R_ bound chromatin strongly during early S-phase, but failed to remain stably associated with replicating chromatin at later stages (Figure 1D, compare ‘lanes 5 and 6’ with ‘lanes 7 and 8’). Thus, the aberrant chromatin recruitment dynamics and reduced chromatin retention of FANCD2_K562R_—and possibly additional functional abnormalities of this mutant—may be caused by defects other than its inability to become monoubiquitinated. Together, these data indicate that FANCD2 mediates chromatin recruitment and foci formation of BLMcx members during normal replication independently of FANCD2^Ub^ formation.

### Defective BLMcx chromatin recruitment in FANCD2-depleted extracts is not caused by replication delay

**Figure 2.**
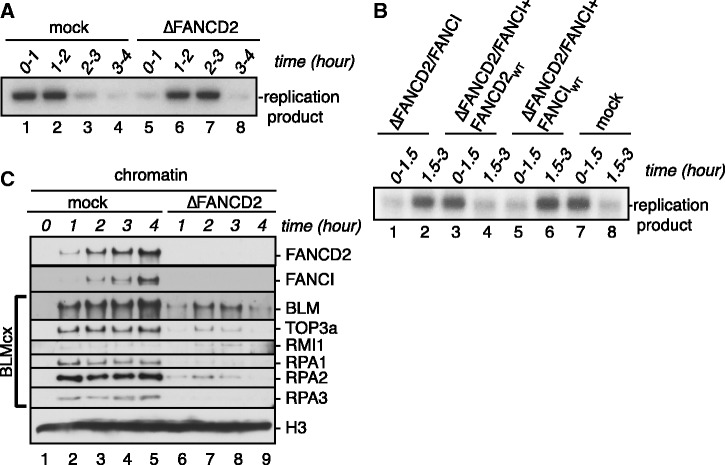
Defective BLMcx chromatin recruitment in FANCD2-depleted extracts is not caused by replication delay. (**A**) FANCD2-depleted S-phase extracts exhibit a delay of replication onset. S-phase extracts were mock depleted (lanes 1–4) or FANCD2 depleted (lanes 5–8). Sperm chromatin was added to extracts and replication was monitored by pulsing replicating extract aliquots with [α-^32^P]dGTP at the indicated time windows. (**B**) FANCD2—but not FANCI—is responsible for timely replication onset. S-phase extracts were mock depleted (lanes 7 and 8), FANCD2/FANCI depleted (lanes 1 and 2) or FANCD2/FANCI depleted and supplemented with myc-FANCD2_WT_ (lanes 3 and 4) or Flag-FANCI_WT_ (lanes 5 and 6). Sperm chromatin was replicated in the different extracts, and replication was monitored by pulsing extract aliquots with [α-^32^P]dGTP at the indicated time windows. (**C**) Parallel to the replication assay shown in Figure 2A, replicating chromatin was re-isolated at indicated time points from mock-depleted extracts (lanes 1–5) or FANCD2-depleted extracts (lanes 6–9), and chromatin fractions were analyzed for bound FANCD2, FANCI and BLMcx members.

Chromosomal replication depends on the presence of RPA (44,45). The fact that FANCD2-depleted extracts showed decreased RPA chromatin loading (Figure 1C and D) raised the question whether replication efficiency was reduced in these extracts. If so, reduced replication efficiency—rather than absence of chromatin-bound FANCD2 *per se*—may interfere with chromatin loading of BLMcx members BLM, RMI1 and TOP3a in FANCD2-depleted extracts. We therefore analyzed efficiency and timing of chromosomal replication in FANCD2-depleted extracts and found that these extracts exhibited a 1-h delay in replication onset (Figure 2A). Adding recombinant FANCD2—but not FANCI—to FANCD2/FANCI double-depleted extracts restored timely replication onset (Figure 2B), indicating that FANCD2 regulates replication onset independently of FANCI. Once initiated, replication progression and efficiency were unaffected in FANCD2-depleted extracts (Figure 2A), supporting recent findings that FANCD2 may be involved in replication initiation but not elongation (46). Because BLMcx binds chromatin in a replication-dependent manner, these results hinted that BLMcx chromatin recruitment might be merely ‘delayed’ in absence of FANCD2. We therefore followed chromatin binding of BLMcx members in mock- and FANCD2-depleted extracts over an extended period (0–4 h instead of 0–2 h in previous assays). Chromatin loading of all BLMcx members was reduced throughout replication and after replication in FANCD2-depleted extracts (Figure 2C). We conclude that physical absence of chromatin-bound FANCD2 *per se*, and not delayed replication onset caused by FANCD2-depletion, is responsible for defective BLMcx chromatin loading.

### FANCD2 mediates DNA damage-induced assembly of BLMcx

**Figure 3.**
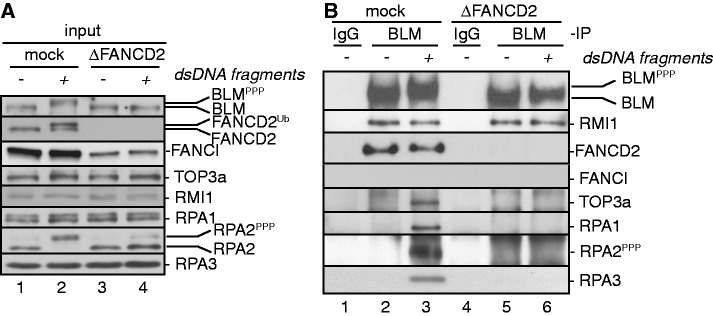
FANCD2 is required for full BLM complex assembly. (**A**) Input panels: S-phase extracts were mock depleted (lanes 1 and 2) or FANCD2 depleted (lanes 3 and 4), and either untreated (lanes 1 and 3) or incubated with dsDNA fragments for 10 min (lanes 2 and 4). (**B**) IP panels: the differently depleted egg extracts described in (A) were subjected to IP with rabbit IgG (lanes 1 and 4) or a Xenopus-specific BLM antibody (lanes 2, 3, 5 and 6). Input and IP samples were analyzed for the presence of FANCD2, FANCI and BLMcx proteins.

In human cells, BLMcx is constitutively present (24). In contrast, BLMcx members BLM and TOP3a do not interact in *Xenopus* S-phase extracts that are initially DNA-free on preparation. Instead, the BLM–TOP3a interaction is inducible by adding a variety of small DNA substrates such as forked DNA structures (mimicking stalled replication forks) or dsDNA fragments (mimicking dsDNA containing DNA DSBs), indicating that complete BLMcx assembly is DNA (damage) dependent (37). We tested if interactions between BLM and other BLMcx members occurred in a DNA-dependent manner as well. BLM and RMI1 interacted in absence or presence of dsDNA fragments, indicating that the BLM/RMI1 complex forms constitutively (Figure 3B). In contrast, interactions of BLM with TOP3a and RPA occurred only in presence of dsDNA fragments, indicating that the full BLM complex does indeed assemble in a DNA (damage)-dependent manner. Interestingly, FANCD2—but not FANCI—co-immunoprecipitated with BLM from DNA-free and DNA-containing extracts indicating that FANCD2 and BLM form a constitutive subcomplex. This subcomplex exists independently of FANCD2^Ub^ formation, as FANCD2 is not monoubiquitinated in DNA-free extracts (Figure 3A) (38,47). To determine if FANCD2 is involved in BLMcx assembly, we analyzed interactions between BLMcx members in mock- or FANCD2-depleted, DNA-free or dsDNA-containing extracts. The interaction between BLM and RMI1 was unaffected in absence of FANCD2, whereas DNA-dependent interactions of BLM with TOP3a and RPA1-3 were blocked (Figure 3B). Thus, full BLMcx assembly occurs only in presence of DNA and is FANCD2 dependent.

### FANCD2 promotes BLM protein stability independently of FANCI

**Figure 4.**
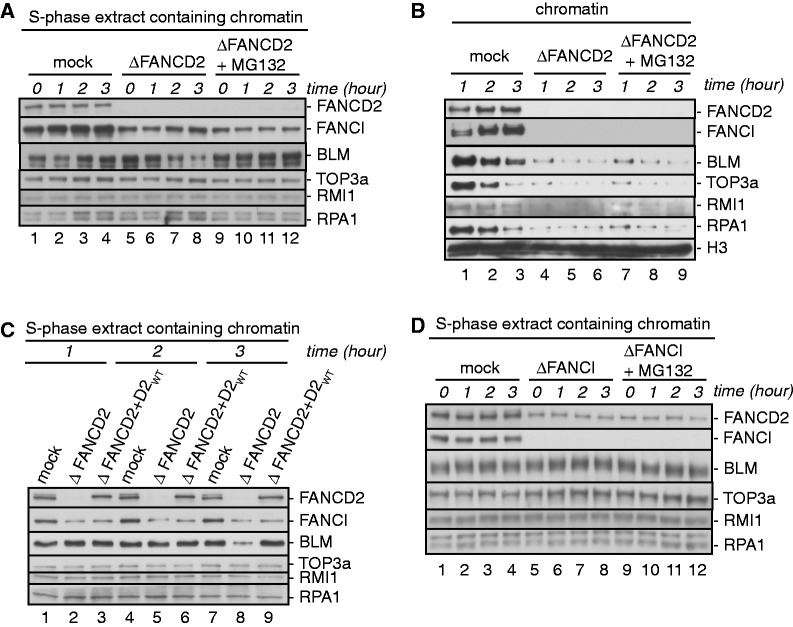
FANCD2 protects BLM protein stability. (**A**) FANCD2 protects BLM protein stability in replicating S-phase extracts. Egg extracts were mock depleted (lanes 1–4), FANCD2 depleted (lanes 5–8) or FANCD2 depleted and treated with MG132 (lanes 9–12). Chromatin was replicated in extracts, and chromatin-containing extract aliquots were taken at indicated time points and analyzed for FANCD2, FANCI and BLMcx members. (**B**) BLM stabilization does not restore BLMcx chromatin binding in FANCD2-depleted extracts. From replicating extracts described in (A), chromatin fractions were isolated at indicated time points and analyzed for chromatin-bound FANCD2, FANCI and BLMcx. (**C**) myc-FANCD2_WT_ restores BLM protein stability in FANCD2-depleted extracts. Egg extracts were mock depleted (lanes 1, 4 and 7), FANCD2 depleted (lanes 2, 5 and 8) or FANCD2 depleted and supplemented with myc-FANCD2_WT_ (lanes 3, 6 and 9). Chromatin was replicated in the extracts and chromatin-containing extract aliquots were taken at indicated time points and analyzed for FANCD2, FANCI and BLMcx members. (**D**) FANCI is dispensable for BLM protein stability. Egg extracts were mock depleted (lanes 1–4), FANCI depleted (lanes 5–8) or FANCI depleted and treated with MG132 (lanes 9–12). Chromatin was replicated in the extracts, and chromatin-containing extract aliquots were taken at indicated time points and analyzed for FANCD2, FANCI and BLMcx members.

The BLMcx member RMI1 interacts directly with BLM and TOP3a and stabilizes both proteins (23,27,48). Because FANCD2 associated with BLMcx, we asked if FANCD2 regulated protein stability of BLMcx members as well. We found that BLM protein levels decreased steadily in FANCD2-depleted replicating extracts, whereas levels of RMI1, TOP3a and RPA remained stable (Figure 4A). Supplementing these extracts with a proteasome inhibitor, MG132, restored BLM protein stability (Figure 4A), indicating proteasome-mediated BLM degradation in absence of FANCD2. Importantly, reconstituting FANCD2-depleted extracts with myc-FANCD2_WT_ stabilized BLM protein levels (Figure 4C), demonstrating that FANCD2 itself (and not a co-depleted protein) maintains BLM protein stability. In contrast, BLM protein levels were unaffected in FANCI-depleted extracts (Figure 4D), suggesting that residual FANCD2 levels (20%) in FANCI-depleted extracts are sufficient to stabilize BLM.

In our previous BLMcx chromatin recruitment assays, we had tested BLMcx protein levels immediately after FANCD2 depletion (Figure 1B), but did not continue to monitor BLMcx protein levels in parallel with the time points (1 and 2 h) at which chromatin samples were isolated from extracts. Our finding that BLM was unstable in absence of FANCD2 raised the possibility that reduced chromatin-bound BLM levels in FANCD2-depleted extracts were due to BLM protein degradation, which in turn would decrease chromatin-bound levels of other BLMcx members as well (31). To investigate this possibility, we analyzed chromatin binding of BLMcx in mock- and FANCD2-depleted extracts in absence and presence of MG132. FANCD2-depleted extracts containing MG132-stabilized, wild-type-like BLM protein levels remained unable to support chromatin recruitment of BLMcx (Figure 4B), demonstrating that FANCD2 does not only promote BLM protein stability, but also functions to recruit BLMcx to replicating chromatin.

### FANCD2 regulates DNA damage-induced phosphorylation of BLM and RPA2 independently of FANCI

**Figure 5.**
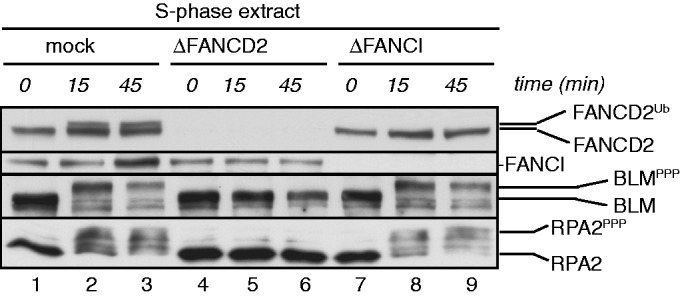
FANCD2 regulates DNA DSB-triggered hyperphosphorylation of BLM and RPA2 independently of FANCI. S-phase egg extracts were mock depleted (lanes 1–3), FANCD2 depleted (lanes 4–6) or FANCI depleted (lanes 7–9), and supplemented with 50 ng/μl dsDNA fragments. Extract aliquots were taken at the indicated time points and analyzed for FANCD2, FANCI, BLM and RPA2.

Following cellular treatment with replication inhibitors or DNA DSB inducers, human BLM and RPA2 are hyperphosphorylated (BLM^PPP^ and RPA2^PPP^; RPA2^PPP^ includes phosphorylation at serine 33) by the checkpoint kinases ATR (Ataxia telangiectasia and Rad3-related) and ATM (Ataxia telangiectasia mutated) (30,33,43,49–51). Similarly, *Xenopus* BLM and RPA2 are hyperphosphorylated in egg extracts containing DNA DSBs [(37) and Figure 5]. Depletion of FANCD2—but not FANCI—from egg extracts completely abrogated DNA DSB-induced BLM^PPP^ and RPA^PPP^ formation (Figure 5), indicating that FANCD2 regulates phosphorylation of BLM and RPA independently of FANCI. Moreover, since monoubiquitination of FANCD2 is blocked in FANCI-depleted extracts (Figure 5), FANCD2^Ub^ formation is dispensable for DNA DSB-induced BLM^PPP^ and RPA^PPP^ formation.

### Regulation of BLMcx by FANCD2 is evolutionarily conserved

**Figure 6.**
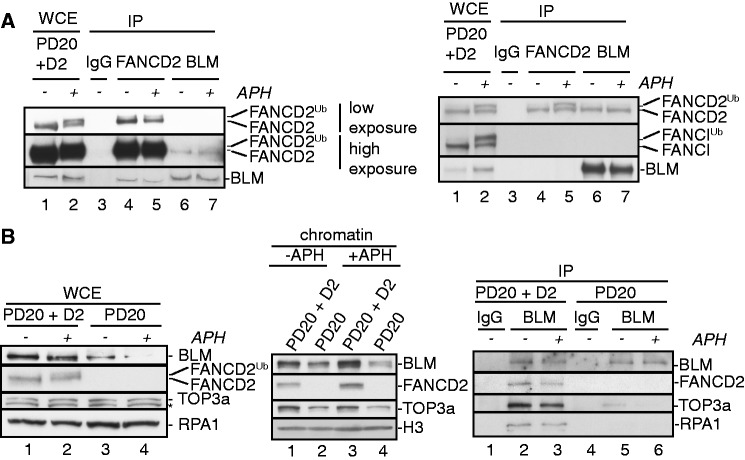
FANCD2-dependent BLM regulation is conserved in human cells. (**A**) Human BLM forms a constitutive complex with non-ubiquitinated FANCD2. Left panel: WCE were prepared from PD20 + D2 cells (lanes 1 and 2) and subjected to IP with rabbit IgG (lane 3), FANCD2 antibody (lanes 4 and 5) or BLM antibody (lanes 6 and 7). Input and equal volumes of IP samples were analyzed for presence of FANCD2 and BLM. Right panel: The same samples shown in the left panel were reanalyzed by extended gel electrophoresis. Sample volumes were adjusted to contain equal amounts of FANCD2 to better discern between non-ubiquitinated and monoubiquitinated FANCD2 isoforms. N.B.: to achieve equal FANCD2 protein concentrations in WCE and IP lanes, 1/30 of the original sample volumes were loaded in lanes 1, 2, 4 and 5. (**B**) Left panel: Human FANCD2 supports BLM protein stability. WCE were prepared from human PD20 + D2 cells (lanes 1 and 2) and PD20 cells (lanes 3 and 4) that had been untreated (lanes 1 and 3) or treated with 30 µM APH (lanes 2 and 4) and analyzed for FANCD2 and BLMcx members. Middle panel: Human FANCD2 mediates BLMcx chromatin recruitment. PD20 + D2 cells (lanes 1 and 3) and PD20 cells (lanes 2 and 4) were untreated (lanes 1 and 2) or treated with 30 µM APH for 3 h (lanes 3 and 4). Chromatin fractions were isolated from the cells and analyzed for presence of FANCD2, BLM and TOP3a. Histone H3: loading control. Right panel: Human FANCD2 is crucial for BLMcx assembly. WCE from untreated or APH-treated PD20 + D2 and PD20 cells were subjected to IP with rabbit IgG (lanes 1 and 4) or BLM antibody (lanes 2, 3, 5 and 6), and IP samples were analyzed for the presence of FANCD2 and BLMcx members.

**Figure 7.**
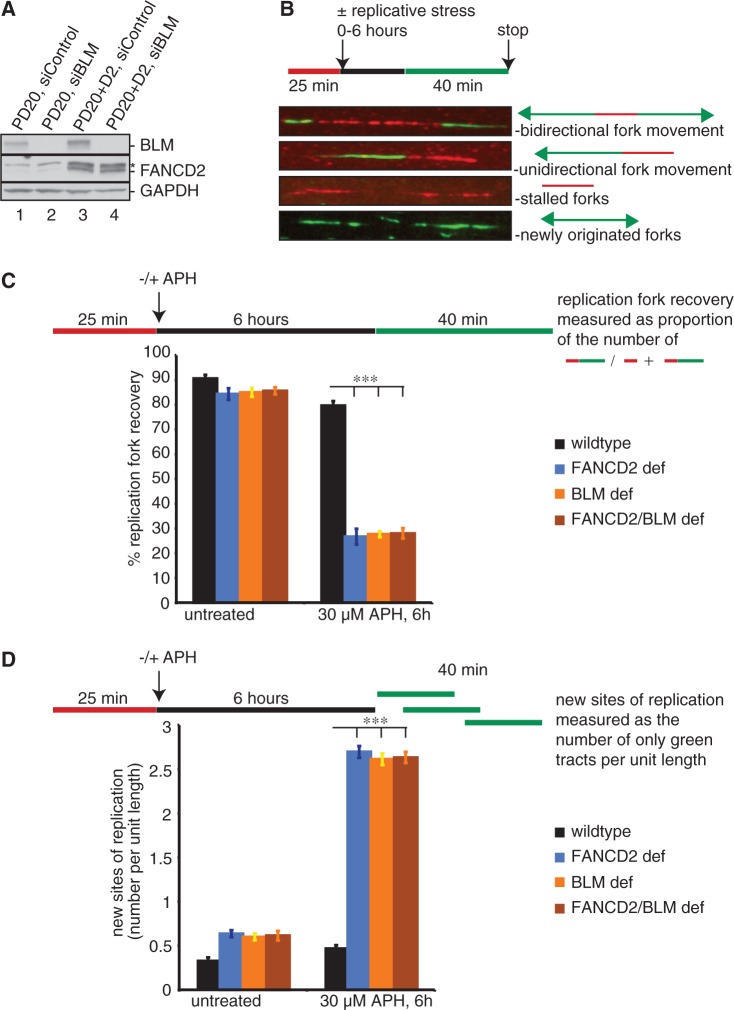
Human FANCD2 and BLM act in one pathway to mediate replication fork restart and suppression of new origin firing. (**A**) Human cell types used for DNA fiber analysis: wild type (PD20 + D2, siControl), FANCD2 deficient (PD20, siControl), BLM deficient (PD20 + D2, siBLM) and FANCD2/BLM double deficient (PD20, siBLM). (**B**) Images of DNA fibers with a schematic of defining sites of replication. Red tracts: DigU; green tracts: BioU. (**C**) FANCD2 and BLM act in a common pathway to mediate replication fork restart after replication blockade. The efficiency of replication restart in wild-type, FANCD2-deficient, BLM-deficient and FANCD2/BLM double-deficient cells was measured as the number of restarted replication forks after APH-mediated fork stalling (DigU→BioU tracts), compared with the total number of DigU-labeled tracts (DigU plus DigU→BioU). (**D**) FANCD2 and BLM act in concert to suppress new origin firing during replication blockade. The number of new sites of replication originating during the 40 min recovery period after APH treatment was compared between wild-type, FANCD2-deficient, BLM-deficient and FANCD2/BLM double-deficient cells. New origins of replication were measured as the number of green-only (BioU) tracts per unit length. ***P < 0.0001.

Our *Xenopus* egg extract-derived results indicated that FANCD2 is a functional regulator of BLMcx that (i) constitutively associates with BLM, (ii) promotes BLM protein stability, (iii) controls BLM chromatin binding and (iv) mediates interactions of BLM with TOP3a and RPA. To test if these functions are conserved in humans, we used the FANCD2-deficient patient fibroblast cell line PD20 and its isogenic complemented counterpart PD20 + D2. As previously described (32,33), we found that a small subpopulation of FANCD2 co-immunoprecipitated with BLM from wild-type (PD20 + D2) cells; moreover, we were able to show the reverse co-immunoprecipitation of cellular BLM with FANCD2 (Figure 6A, left panel). The FANCD2-BLM interaction occurred regardless of the presence or absence of the replication inhibitor aphidicolin (APH), a potent inducer of FANCD2^Ub^ formation (Figure 6A), hinting that this interaction does not require FANCD2 monoubiquitination. To further investigate this, we reanalyzed the samples shown in Figure 6A (left panel) by comparing migration patterns of FANCD2 isoforms between WCEs, FANCD2 immunoprecipitates and BLM immunoprecipitates that were volume-adjusted to contain ‘equal amounts of FANCD2’. As shown in Figure 6A (right panel), the anti-FANCD2 antibody immunoprecipitated both FANCD2 isoforms, whereas the anti-BLM antibody co-immunoprecipitated predominantly non-ubiquitinated FANCD2 even from APH-treated PD20 + D2 cells. Thus, human FANCD2 and BLM interact constitutively in a DNA damage- and FANCD2^Ub^-independent manner, mirroring the behavior of their *Xenopus* homologs (see Figure 3). In addition, the human FANCD2–BLM complex did not contain FANCI (Figure 6A, right panel), similar to our observation in *Xenopus* extracts (see Figure 3). Further analysis revealed that BLM protein levels—but not TOP3a or RPA protein levels—were reduced in PD20 cells compared with PD20 + D2 cells and this phenotype became more pronounced in response to cellular treatment with APH (Figure 6B, left panel; also shown in Figure 7A). Consequently, chromatin-bound BLM and TOP3a protein levels were reduced in untreated or APH-treated PD20 cells compared with PD20 + D2 cells (Figure 6B, middle panel). Lastly, we tested if human BLMcx formation was FANCD2-dependent. Importantly, despite lower BLM levels in PD20 cells (Figure 6B, left panel), we were able to immunoprecipitate equal BLM protein amounts from PD20 and PD20 + D2 cells (Figure 6B, right panel). Strikingly, BLM immunoprecipitates from PD20 cells contained significantly less TOP3a and RPA than those from PD20 + D2 cells, indicating that complete BLMcx assembly is indeed FANCD2-dependent in human cells. Together, these findings indicate that FANCD2 regulates BLMcx functions in both frogs and humans.

### FANCD2 and BLM act in concert to restart stalled replication forks

Human BLM mediates replication fork recovery following cellular treatment with replication inhibitors such as APH or hydroxyurea (HU). In BS cells, <40% of all stalled replication forks are able to restart and consequently, global replication is rescued by new origin firing (30). BLMcx members RMI1 and RPA also contribute to replication fork restart (31,43), indicating that BLMcx acts as functional entity to support replication fork recovery. Because our results described above showed that *Xenopus* FANCD2 regulates the BLM pathway in S-phase, we asked if human FANCD2 was essential for BLM-mediated replication fork restart. For our assay, we used PD20 and PD20 + D2 cells. In addition, we generated BLM-deficient and FANCD2/BLM double-deficient cells via siRNA-mediated BLM knockdown in PD20 + D2 cells and PD20 cells, respectively (Figure 7A). Replication events on individual chromosomes were monitored with a dual-labeling DNA fiber assay. Cells were pulsed with DigU (red label) for 25 min, then untreated or treated with 30 µM APH for 6 h, followed by a pulse with BioU (green label) for 40 min (Figure 7B). Because stalled replication forks are progressively inactivated over time (52), we first confirmed that the wild-type-like PD20 + D2 cells used in our study were able to restart the majority of stalled replication forks (∼80%), and did not significantly upregulate new origin firing after 6 h of APH-treatment, comparable with other wild-type cells treated with APH or HU for 1–6 h (Supplementary Figure S4A and B) (30,52). Strikingly different from efficient fork restart in the wild-type cells, the proportion of replication forks competent for restart was severely—and equally—reduced in FANCD2 and BLM-deficient cells (26.6 and 27.6%, *P* < 0.0001). Moreover, this defect in replication restart was not further exacerbated in FANCD2/BLM double-depleted cells (28.6%, *P* < 0.0001) (Figure 7C), indicating that FANCD2 and BLM cooperate to restart replication forks. In parallel, the proportion of newly originated replication tracts (BioU label only) increased significantly and equally (∼5-fold; *P* < 0.0001) in FANCD2-, BLM- and FANCD2/BLM double-deficient cells compared with wild-type cells (Figure 7D). In a reverse approach, siRNA-mediated knockdown of FANCD2 in a BLM-deficient patient cell line, GM08505 (Supplementary Figure S5A), exacerbated neither the level of stalled replication forks nor the upregulation of newly fired origins observed in these cells (Supplementary Figure S5B and C) (30). These data indicate that FANCD2 and BLM act in a common pathway to mediate replication fork restart and to suppress firing of new replication origins following APH-triggered fork stalling.

**Figure 8.**
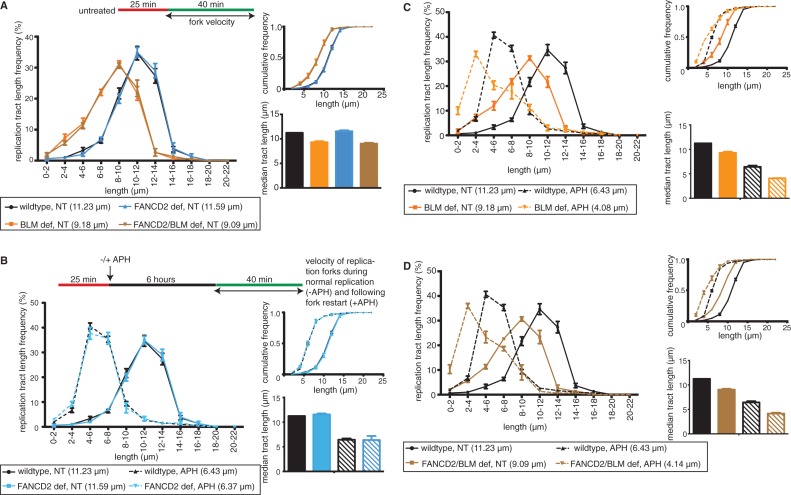
Human BLM acts independently of FANCD2 to maintain velocity of restarted replication forks after APH treatment. (**A**) BLM maintains replication fork velocity in unperturbed conditions independently of FANCD2. BioU tract length distributions were determined on DigU→BioU double-labeled DNA fibers isolated from untreated (NT) wild-type, FANCD2-deficient, BLM-deficient or FANCD2/BLM double-deficient cells. (**B–D**) BLM maintains replication fork velocity of restarted forks independently of FANCD2. BioU tract length distributions were determined on double-labeled DNA fibers before (NT) and after APH treatment and compared between wild-type and (B) FANCD2-deficient cells, (C) BLM-deficient cells and (D) FANCD2/BLM double-deficient cells. For A–D, median tract lengths are indicated below each panel. Insets: Cumulative distributions (top) and plotted median tract lengths (bottom).

In addition to its role in replication fork recovery, BLM maintains replication fork velocity in unperturbed S-phase (53) and following replication fork stalling (2). We asked if human FANCD2 was required for these functions. Replication fork progression was analyzed by measuring BioU tract lengths on DigU→BioU double-labeled tracts. Replication speed was comparable between wild-type and FANCD2-deficient cells (Figure 8A, 11.23 µm and 11.59 µm; *P* = 0.3392). In contrast, replication tracts were shorter in BLM-deficient and BLM/FANCD2 double-deficient cells (Figure 8A, 9.18 and 9.09 µm; *P* < 0.001), indicating that BLM—but not FANCD2—maintains replication speed in normal conditions.

Next, we asked if the velocity of ‘restarted’ replication forks depended on FANCD2 and/or BLM. Importantly, as most APH-stalled forks cannot restart in absence of FANCD2 or BLM (Figure 7C and Supplementary Figure S5B), the majority of replication tracts formed after APH treatment stem from newly originated forks in FANCD2 or BLM-deficient cells. However, analyzing BioU replication tract lengths selectively on DigU→BioU double-labeled fibers after APH treatment allowed us to distinguish restarted forks from newly originated forks. The length of restarted replication tracts was significantly shorter than replication tract lengths in untreated conditions, but equally so in wild-type and FANCD2-deficient cells [Figure 8B, wild type: 11.23 → 6.43 µm (43% reduction); FANCD2 deficient: 11.59 → 6.37 µm (45% reduction). In contrast, BLM- and FANCD2/BLM double-deficient cells that already presented with shorter replication tracts in untreated conditions (see Figure 8A), exhibited even further shortening of restarted replication tracts [Figure 8C, BLM deficient: 9.18 → 4.08 µm (66% reduction); Figure 8D, FANCD2/BLM double deficient: 9.09 → 4.14 µm (65% reduction)]. Thus, human BLM maintains the velocity of normally progressing forks and of restarted replication forks independently of FANCD2.

Supported by the fact that FANCD2-depleted cells allowed residual BLM chromatin binding (Figure 6B, middle panel), this finding indicated that the BLM pathway retains partial functionality in absence of FANCD2. Interestingly, the reduced replication fork velocity in BLM-deficient cells is caused by a severe pyrimidine pool imbalance due to significantly reduced cytidine deaminase (CDA) expression levels (54). We found that CDA protein levels were diminished in BLM-deficient cells but normal in FANCD2-deficient cells (Supplementary Figure S6), indicating that BLM-dependent CDA expression—and thus maintenance of replication fork speed—remains functional in absence of FANCD2.

**Figure 9.**
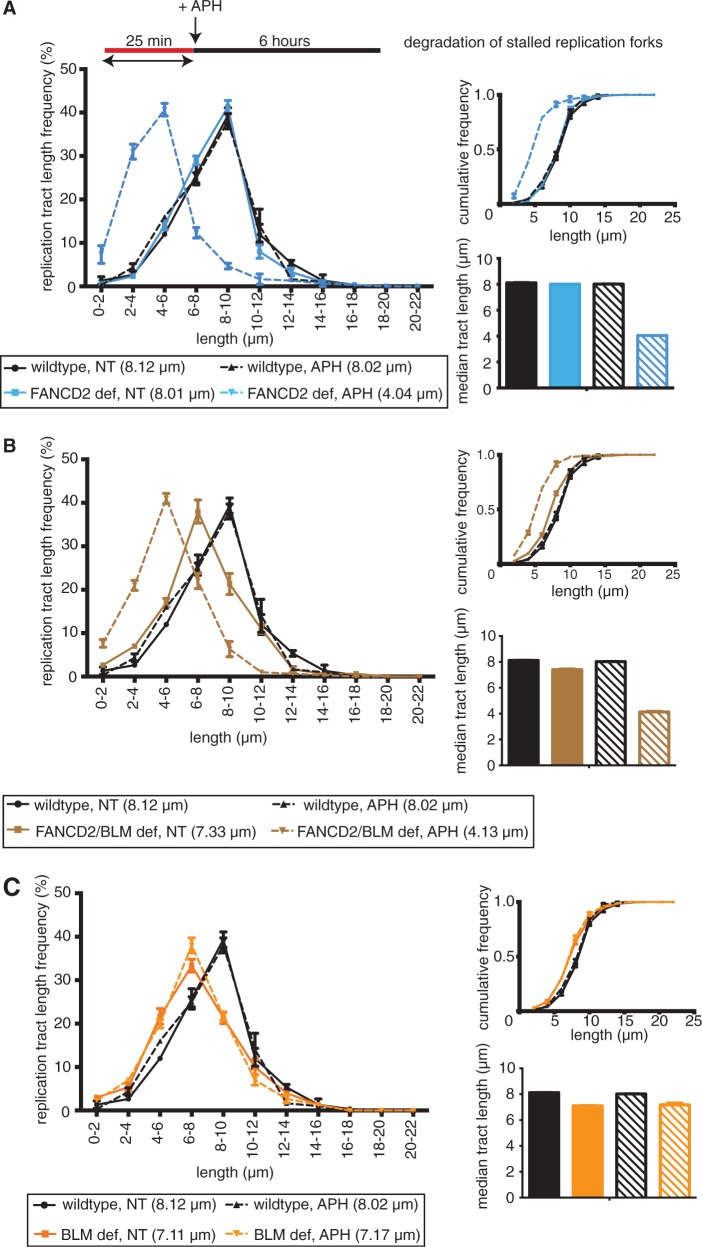
Human FANCD2 acts independently of BLM to protect replication forks from degradation. Lengths of nascent replication fork tracts indicating fork stability (labeled with DigU only) were measured before (NT) and after 6 h of APH treatment. Preformed DigU tract lengths shorten during APH-treatment in (**A**) FANCD2-deficient (PD20) cells compared with wild-type (PD20 + D2) cells and in (**B**) FANCD2/BLM double-deficient (PD20, siBLM) cells compared with wild-type (PD20 + D2) cells. (**C**) Preformed DigU tract lengths do not shorten during APH-treatment in BLM-deficient (PD20 + D2, siBLM). Median tract lengths are indicated below each panel. Insets: Cumulative distributions (top) and plotted median tract lengths (bottom).

Intriguingly, a recent study reported that FANCD2—but not BLM—acted to protect nascent DNA strands at stalled replication forks against degradation by the MRE11 nuclease (2). The authors used single-label DNA fiber analysis to determine the length of already replicated tracts before and after HU treatment in FANCD2-deficient human cells and BLM-deficient mouse embryonic stem cells. In support of this study, we observed a significant difference in DigU-labeled replication tract lengths before versus after APH treatment in human FANCD2-deficient cells (Figure 9A, 8.01 and 4.04 µm; *P* < 0.001) as well as in FANCD2/BLM double-deficient cells (Figure 9B, 7.33 and 4.13 µm; *P* < 0.001). In contrast, DigU tract lengths were comparable before versus after APH-treatment in wild-type cells (Figure 9C, 8.12 and 8.02 µm; *P* = 0.5386) and BLM singly deficient cells (Figure 9C, 7.11 and 7.17 µm; *P* = 0.8463). Thus, FANCD2 protects stalled replication forks independently of BLM in human cells.

In summary, our results demonstrate that the human FANCD2 and BLM proteins have partially overlapping functions during replication fork stalling: they act in concert to mediate replication fork recovery and to suppress new origin firing; at the same time, FANCD2 acts independently of BLM to protect stalled replication forks from degradation, whereas BLM functions independently of FANCD2 to support normal replication speed on fork restart.

## DISCUSSION

Accumulating evidence indicates that functional interactions of upstream and downstream FA pathway proteins with BLMcx members promote repair of replication-stalling DNA lesions. Adding another piece to the emerging puzzle of the FA/BLM pathway, we show that the central FANCD2 protein is an essential regulator of BLMcx and acts in concert with BLM to promote replication fork recovery. In addition, we show that FANCD2’s dimerization partner FANCI is not involved in controlling BLMcx, demonstrating functional separation of FANCD2 from FANCI.

Our results indicate that FANCD2 controls BLMcx on several levels: it protects BLM protein stability and mediates formation, chromatin recruitment and DNA damage-triggered phosphorylation of BLMcx. Importantly, these functions are essentially conserved between *Xenopus* and humans. FANCD2 shares its role as BLM stabilizer with RMI1 and FANCJ, both of which interact directly with BLM, hinting that FANCD2 and BLM may physically interact as well. Our discovery that FANCD2 mediates BLM complex assembly is unexpected because all hitherto known BLMcx members are highly conserved, whereas no FANCD2 homologs exist in lower organisms. Moreover, most BLMcx members interact directly *in vitro* (55) without relying on accessory factors. However, BLM complex assembly may be more tightly regulated *in vivo* and involve helper proteins such as FANCD2.

Why did original cell-based studies not identify FANCD2 as BLMcx member? One reason may be that only a subset of FANCD2 molecules is complexed with BLM both in *Xenopus* and humans. Nevertheless, two recent studies reported FANCD2 to co-immunoprecipitate with BLM from human cells (32,33). While our findings support these studies regarding the existence of a BLM–FANCD2 complex, they simultaneously oppose another conclusion made by both studies that BLM interacts only with FANCD2^Ub^. This conclusion was based on gel electrophoresis results showing co-migration of the FANCD2 isoform present in BLM immunoprecipitates with FANCD2^Ub^ in WCEs. In our hands, however, the migration patterns of FANCD2 isoforms differ between IP and WCE samples. In regular gel electrophoresis, immunoprecipitated ‘non-ubiquitinated’ FANCD2 migrates at a similar rate as FANCD2^Ub^ in WCEs (see Figure 6A, left panel). This can be overcome by extended electrophoresis times: here, a comparison of FANCD2 isoforms between WCEs and directly (anti-FANCD2) or indirectly (anti-BLM) immunoprecipitated samples reproducibly shows that human BLM preferably pulls down the non-ubiquitinated isoform of FANCD2 (Figure 6A, right panel).

Previous studies disagreed whether FANCD2 recruited BLM to sites of DNA damage (32,33). Our findings suggest an essential role for FANCD2 in recruiting the entire BLM complex to chromatin even in undamaged conditions. Interestingly, recruitment of human BLM to replication stress-induced chromatin foci also depends on FANCM (3), hinting that FANCM and FANCD2 cooperate to recruit BLMcx to stalled replication forks.

Beyond assembly and recruitment of BLMcx to chromatin, FANCD2 also regulates the ATR/ATM-dependent phosphorylation of BLMcx members BLM and RPA2, at least in *Xenopus*. Combined with results from the Gautier laboratory that FANCD2 regulates DNA ICL-induced Chk1 phosphorylation (34), these data indicate a broader role for FANCD2 in mediating phosphorylation events during the DNA damage response. Phosphorylation of human BLM at threonine 99 is crucial for its role in replication restart (30), and similarly RPA2 phosphorylation at serine 33 promotes replicative DNA synthesis after fork stalling (43). Thus, FANCD2 may modulate those BLMcx phosphorylation events that contribute to successful fork restart.

Remarkably, FANCI is not part of the BLM complex and dispensable for its chromatin recruitment and phosphorylation, supporting our recently proposed model of distinct roles for FANCD2 and FANCI based on dissociation of the FANCD2/FANCI heterodimer on FA pathway activation (38). Unfortunately, confirming these findings in human cells is complicated by the fact that FANCD2 is unstable in FANCI-deficient cell lines (6), hampering the usefulness of these cells in discerning functions of FANCI from those of FANCD2.

Because FANCI is crucial for FANCD2 monoubiquitination (6,38), our findings also demonstrate that FANCD2^Ub^ formation is dispensable for BLMcx regulation. Nevertheless, chromatin-bound FANCD2 is predominantly in its monoubiquitinated state (36,56). We thus predict that FANCD2 is a multitasking molecular platform, coordinating proteins like BLMcx that dock onto FANCD2 regardless of its monoubiquitination status with those that exclusively bind FANCD2^Ub^ such as FAN1 or SLX4. Notably, a number of previous studies predicted monoubiquitination of FANCD2 to be essential for all of its functions because the non-ubiquitinatable FANCD2 mutant (human FANCD2_K561R_; *Xenopus* FANCD2_K562R_) was not detectable on chromatin and did not rescue the FA phenotype of FANCD2-deficient cells. Our observation that non-ubiquitinated FANCD2_WT_ binds chromatin normally while FANCD2_K562R_ exhibits different chromatin-binding dynamics and fails to remain stably associated with chromatin over time, hints that at least some of this mutant’s defects are caused by factors other than absence of an ubiquitin moiety at the target lysine (e.g. structural abnormalities). Thus, caution should be used when predicting monoubiquitination-dependent FANCD2 functions based solely on protein behavior of this particular FANCD2 mutant.

Interestingly, a subpopulation of BLMcx members binds chromatin independently of FANCD2, and in this regard, FANCD2-deficient cells differ from BLM-deficient cells in that they maintain appropriate replication fork velocity. In striking contrast, FANCD2 and BLM-deficient cells exhibit identical defects in replication restart indicating that merely ‘reduced’ BLM chromatin loading interferes with replication fork recovery.

Our discovery that FANCD2 and BLM cooperate to achieve replication restart seemingly contradicts conclusions made in a recent study by Schlacher *et al.* (2012). These authors proposed that FANCD2 (in concert with BRCA2 and RAD51) protected stalled replication forks from MRE11-mediated degradation, but was dispensable for replication fork restart and thus functionally distinct from BLM. However, this is likely due to experimental differences between the two studies: We used the same single-label DNA fiber assay as Schlacher *et al.* to confirm that FANCD2—but not BLM—protects stalled replication forks against degradation in human cells (Figure 9). However, to analyze replication fork restart, we used dual DNA fiber labeling (30) that distinguishes between stalled, restarted and newly originated replication forks and allows to determine actual fork restart efficiencies. In contrast, Schlacher *et al.* used single (second) labeling of DNA fibers that assesses the velocity of moving replication forks (restarted and newly originated forks combined) and interpreted the fact that overall replication fork velocity after HU treatment was reduced in BLM- but not in FANCD2-deficient cells as indicator that FANCD2 was not involved in replication fork restart *per se*. Our results indicate a different scenario where FANCD2 and BLM coordinate their functions to promote replication fork restart while suppressing new origin firing; at the same time, FANCD2 and BLM have non-overlapping roles in protecting replication forks from degradation and in promoting the velocity of restarted forks, respectively.

Consequently, we suggest a new extended FA pathway model that encompasses the dual role of FANCD2 at stalled replication forks: (i) regulation of the BRCA2 pathway to protect stalled forks from degradation (2), and (ii) regulation of the BLM helicase pathway to mediate replication fork restart while preventing initiation of new replication tracts (Supplementary Figure S7).

## SUPPLEMENTARY DATA

Supplementary Data are available at NAR Online: Supplementary Table 1 and Supplementary Figures 1–7.

## FUNDING

National Science Foundation [award 1121023]; American Cancer Society [RSG-13-039-01-DMC]; Leukemia Research Fund (Masonic Cancer Center). I.C. was funded by the American Heart Association. Funding for open access charge: National Science Foundation [award 1121023].

*Conflict of interest statement.* None declared.

## Supplementary Material

Supplementary Data
