# Supplementary material for: FANCD2 regulates BLM complex functions independently of FANCI to promote replication fork recovery
Source: Nucleic Acids Res. 2013 May 8;41(13):6444–59. doi: 10.1093/nar/gkt348 (PMC3711430; doi:10.1093/nar/gkt348)
Supplement: Supplementary Data [file supp_41_13_6444__index.html]

FANCD2 regulates BLM complex functions independently of FANCI to promote replication fork recovery — Supplementary Data 

# FANCD2 regulates BLM complex functions independently of FANCI to promote replication fork recovery

## Supplementary Data

files

**Files in this Data Supplement:**

- Supplementary Data - pdf file
