# Supplementary material for: FANCD2 regulates BLM complex functions independently of FANCI to promote replication fork recovery
Source: Nucleic Acids Res. 2013 May 8;41(13):6444–59. doi: 10.1093/nar/gkt348 (PMC3711430; doi:10.1093/nar/gkt348)
Supplement: Supplementary Data [file supp_gkt348_Supplementary_info.pdf]

Chaudhury et al.

Coomassie blue staining of the polyacrylamide gel showing purified recombinant *Xenopus laevis* wildtype and ubiquitination dead mutant FANCD2 proteins

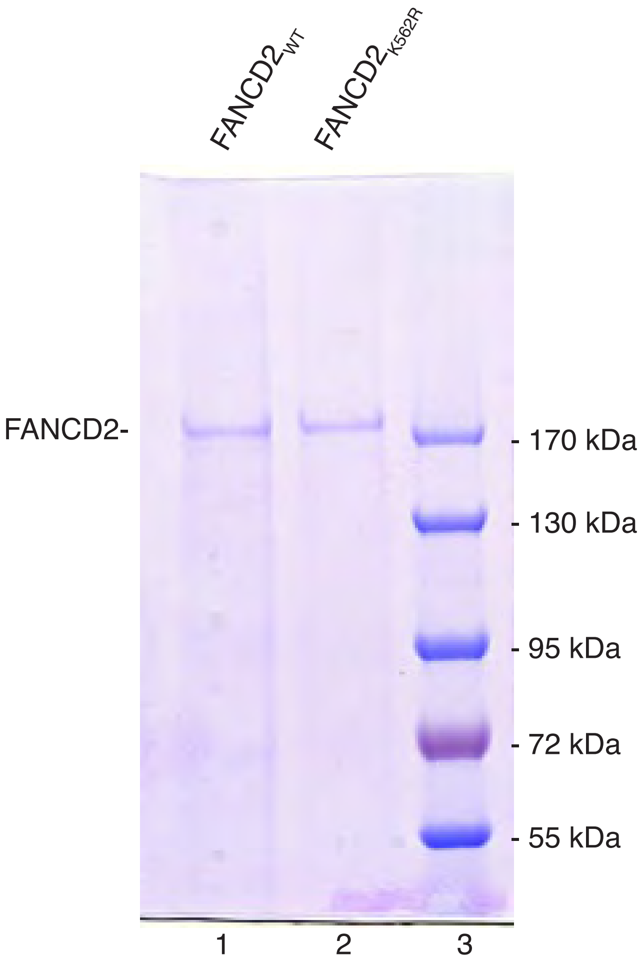

Legend: Recombinant *Xenopus laevis* wildtype ($FANCD2_{WT}$, lane 1) and ubiquitination dead mutant ($FANCD2_{K562R}$, lane 2) FANCD2 proteins were expressed in Sf9 insect cells and purified using nickel resin. Protein molecular weight marker is shown in lane 3.

BLMcx recruitment to replicating chromatin is essentially unaffected in FANCI-depleted extracts

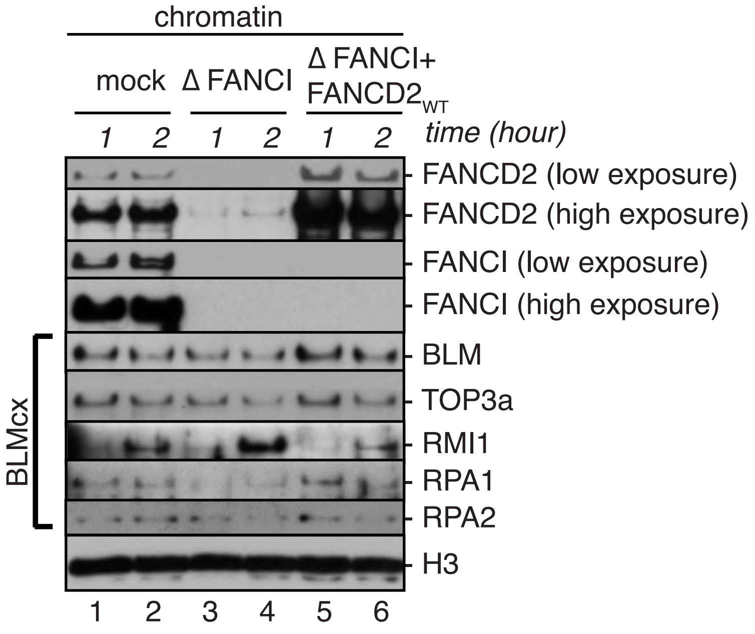

Legend: S-phase extracts were mock-depleted (lanes 1 and 2), FANCI-depleted (lanes 3 and 4) or FANCI-depleted and reconstituted with myc–FANCD2$_{WT}$ (lanes 5 and 6). Sperm chromatin was added to the extracts and allowed to replicate. Chromatin was reisolated at the indicated time points, and analyzed for bound FANCD2, FANCI and BLMcx members.

Figure S 3

Chaudhury et al.

FANCD2 regulates foci formation of BLM and RPA2^{S33-P} during late S-phase

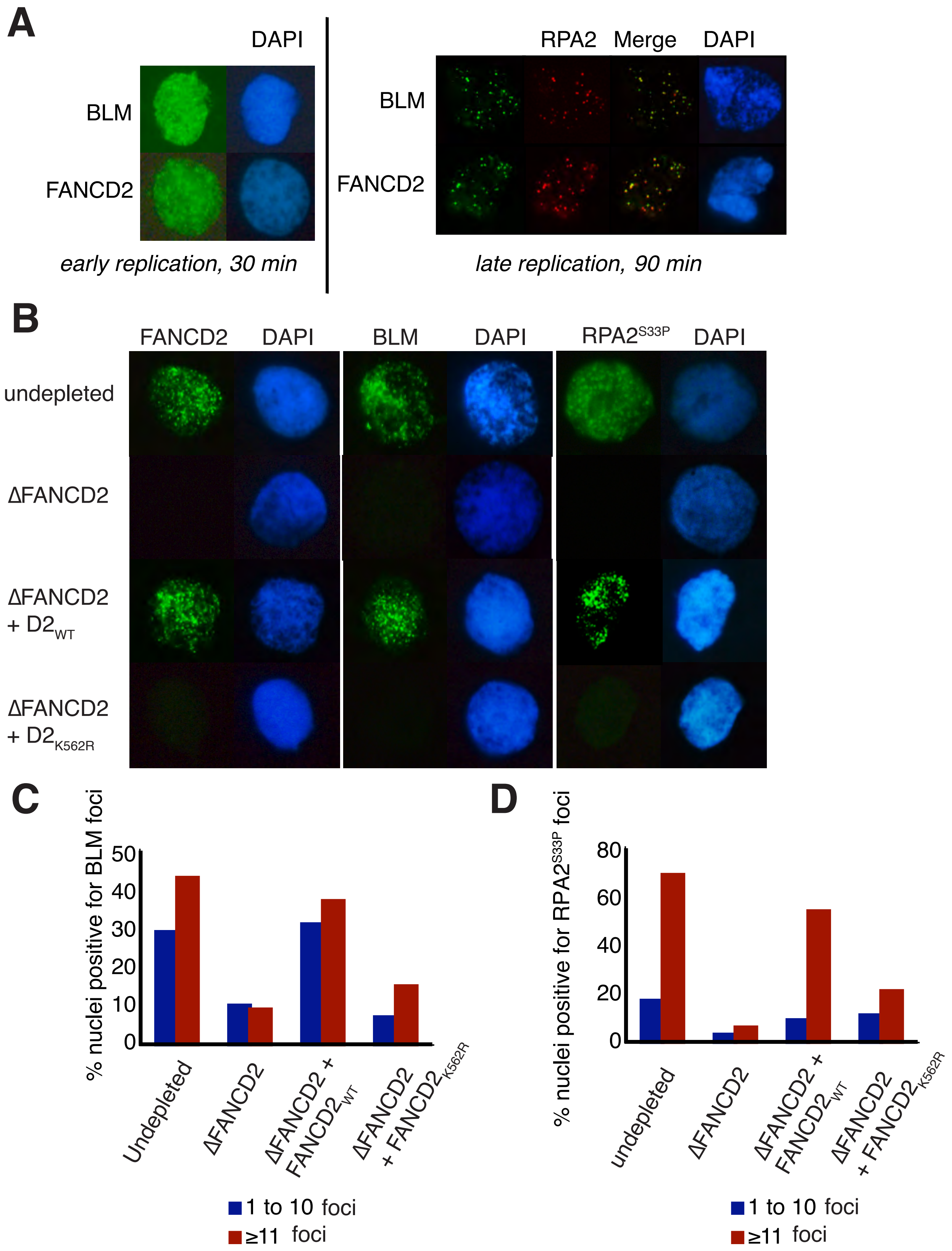

Legend: ***(A)*** *BLM, RPA2 and FANCD2 colocalize in nuclear foci during late S-phase*. Sperm nuclei were replicated in *Xenopus* S-phase extracts and reisolated at 30 min (mid-replication) and 90 min (late replication) and analyzed for the presence of FANCD2, BLM and RPA2 by immuno-fluorescence analysis. N.B.: We were not able to show colocalization of FANCD2 and BLM foci since the available *Xenopus*-specific antibodies against both proteins were generated in rabbits.

Figure S 3 continued

***(B)** FANCD2$_{WT}$, but not FANCD2$_{K562R}$ rescues BLM and RPA2^{S33-P} foci formation in FANCD2-depleted extracts*. Sperm nuclei were replicated in S-phase extracts that were undepleted, FANCD2-depleted, or FANCD2-depleted and supplemented with recombinant FANCD2$_{WT}$ or FANCD2$_{K562R}$. Nuclei were isolated at 90 min and analyzed for foci formation of BLM and RPA2^{S33-P}.
(C and D) Histogram of quantitative analysis of nuclei containing BLM **(C)** and RPA2^{S33-P} **(D)** foci in the differently depleted extracts described in **(B)**. Nuclei were tabulated in groups containing 0–10 and ≥ 11 foci per nucleus.

Figure S 4

Chaudhury et al.

PD20+D2 and GM00637 cells exhibit normal replication restart responses after 6 h treatment with APH or HU

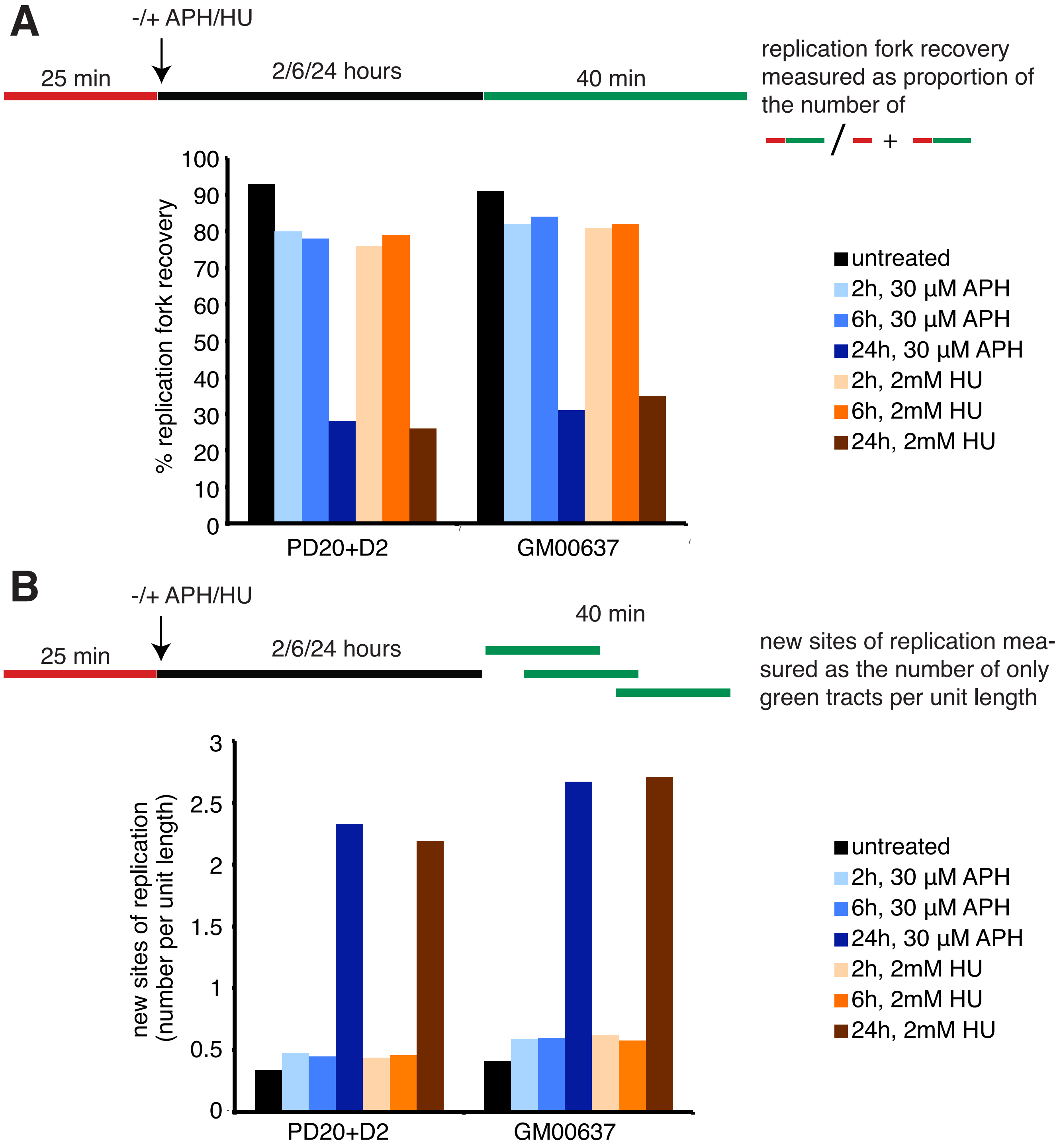

Legend: PD20+D2 (wildtype control for PD20) cells and GM00637 (wildtype control for GM08505) cells were either untreated or treated with APH (30 µM) or HU (2mM) for 2, 6 or 24 hours. ***(A)*** The efficiency of replication restart was measured as the number of restarted replication forks after APH or HU-mediated fork stalling (DigU→BioU tracts), compared to the total number of DigU-labeled tracts (DigU plus DigU→BioU tracts). 80% of replication forks restarted in PD20+D2 and GM00637 cells following APH or HU-mediated stalling for 2 h and 6 h, but not 24 h. ***(B)*** The new sites of replication visible during the 40 min recovery period after APH or HU treatment were measured as the number of green-only (BioU) tracts per unit length. New origin firing was suppressed in PD20+D2 and GM00637 cells after APH- or HU-mediated replication fork stalling for 2 h and 6 h, but not 24 h.

Figure S 5

Chaudhury et al.

siRNA-mediated knockdown of FANCD2 in a BLM-deficient patient cell line, GM08505, does not exacerbate defects in replication fork restart or in suppression of new origin firing

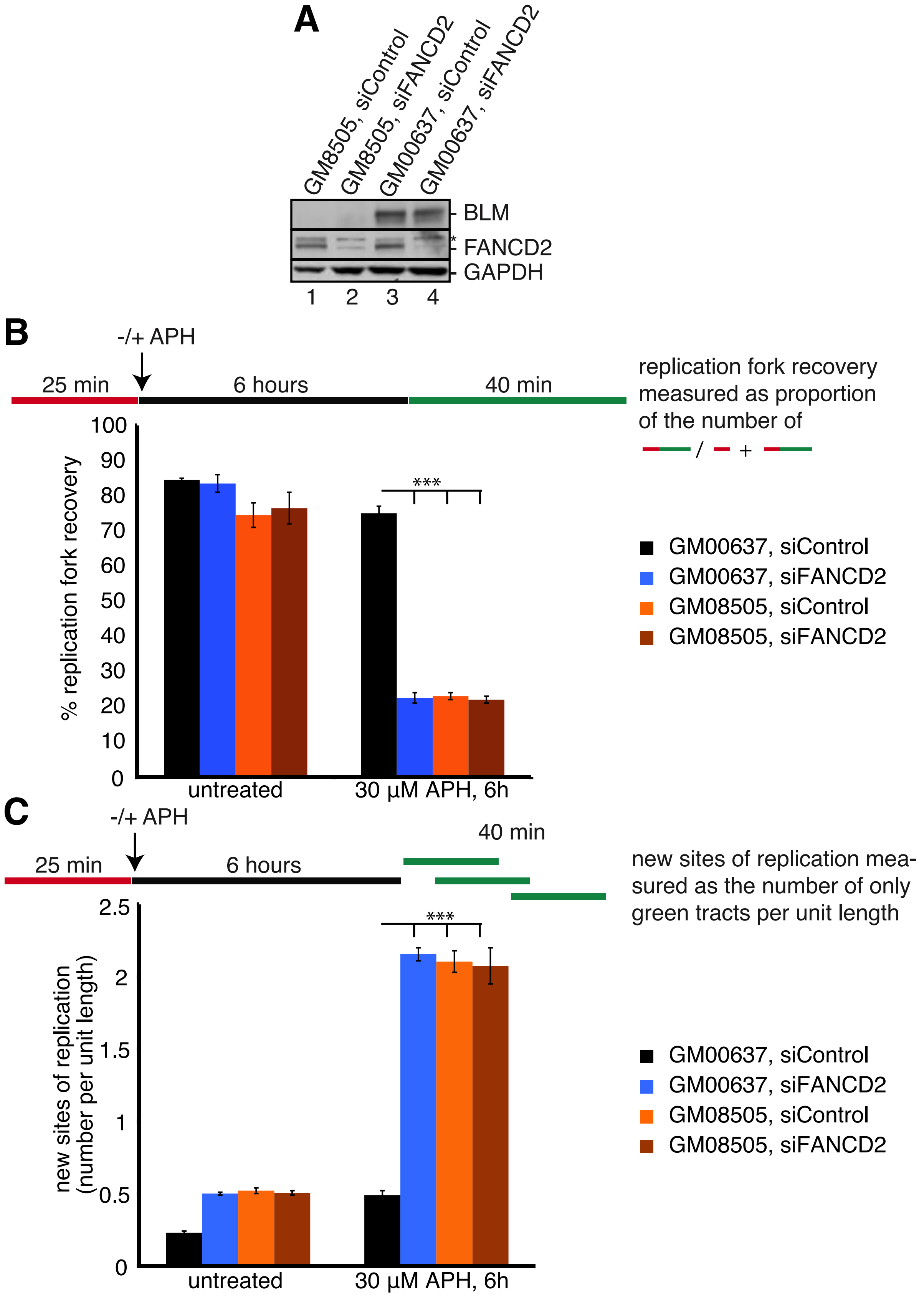

***<u>Legend:</u>* *(A) Cell types used in panels B and C*: wildtype (GM00637, siControl), FANCD2-deficient (GM00637, siFANCD2), BLM-deficient (GM08505, siControl) and FANCD2/BLM double-deficient (GM08505, siFANCD2).

Figure S 5 continued

(B) *FANCD2 depletion does not further reduce replication fork recovery in GM08505 cells*. The efficiency of replication restart was measured as the number of restarted replication forks after APH-mediated fork stalling (DigU→BioU tracts), compared to the total number of replication forks (DigU plus DigU→BioU tracts). ***(C)*** *FANCD2 depletion does not increase new origin firing in GM08505 cells*. The new sites of replication visible during the 40 min recovery period after APH treatment were measured as the number of green-only (BioU) tracts per unit length. ***, P < 0.0001.

Figure S 6

Chaudhury et al.

Cytidine deaminase (CDA) protein levels are stable in FANCD2-deficient cells

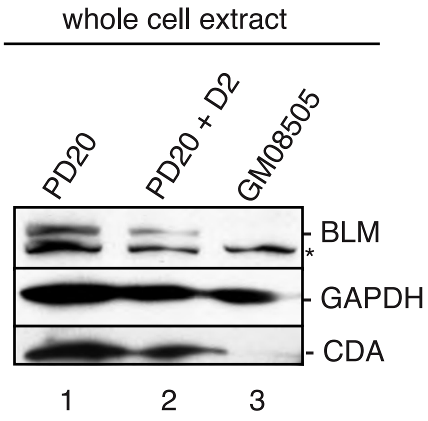

Legend: Whole cell extracts were prepared from FANCD2-deficient cells (PD20, lane 1), wild-type cells (PD20+D2, lane 2) and BLM-deficient cells (GM08505, lane 3) and analyzed for the presence of BLM and CDA. GAPDH: loading control.

Figure S 7

Chaudhury et al.

Model of a dual role for FANCD2 in protecting replication fork stability and mediating replication fork restart

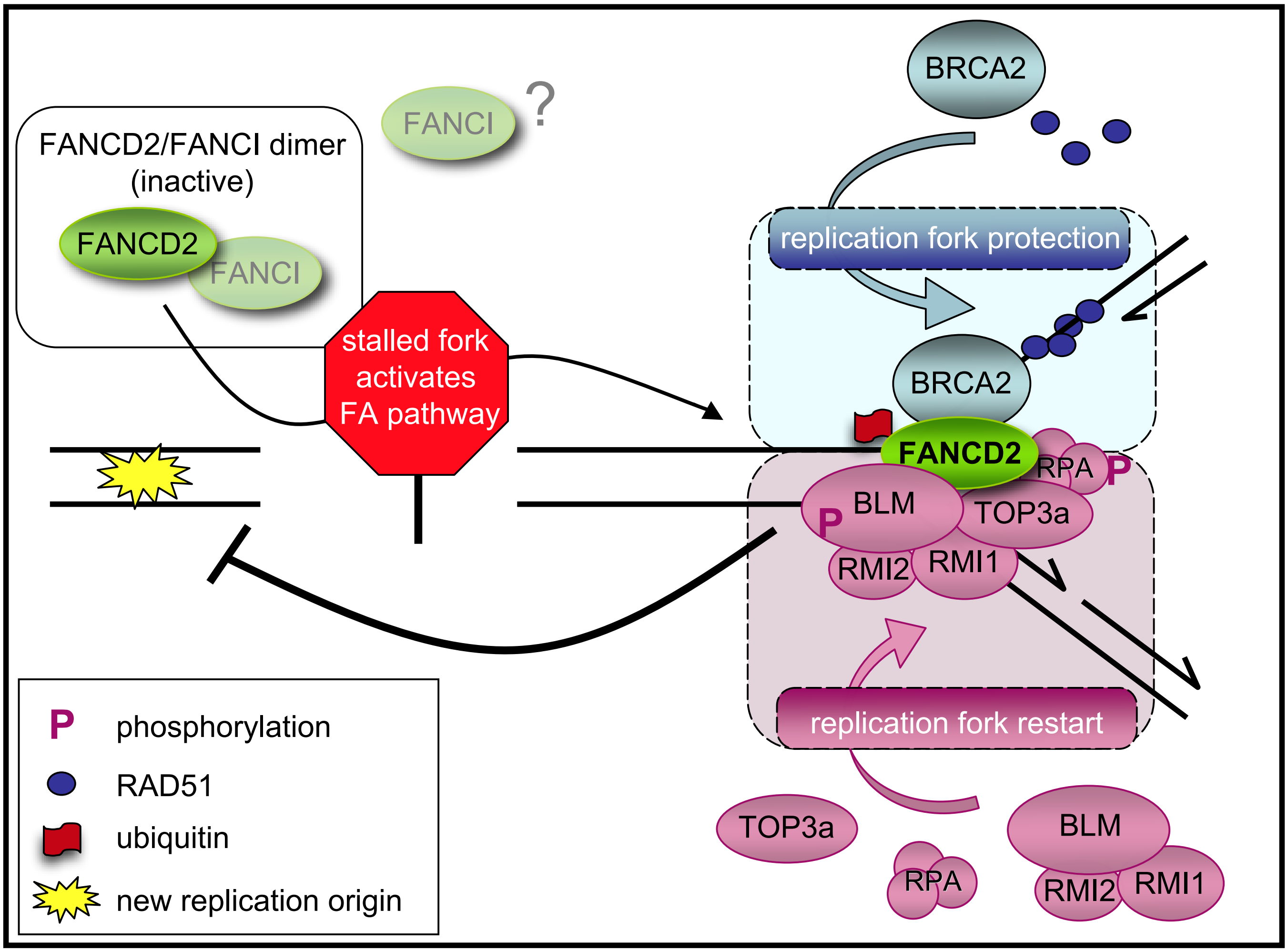

Legend: Following replication fork stalling, FANCD2 dissociates from FANCI (38) and is recruited to the stalled fork. Here, FANCD2 fulfills two distinct functions: (a) it recruits BRCA2 and stabilizes RAD51 at the fork to prevent fork degradation (2) and (b) it assembles the BLM complex at the fork and mediates BLMcx phosphorylation to promote replication fork restart. Simultaneously, FANCD2 and BLM act in concert to suppress firing of new replication origins.

Table S 1. DNA fiber data analysis for Figs. 8 and 9.

Cell line	Treat-ment	Fiber analyzed	# fibers	# exper-iments	Median total	Mean of medians	* p-value (two-tailed)	Figure #
1. Wild type	media	Bio-dUTP	900	3	11.24	11.23	0.3392 vs. 2<br><0.0001 vs. 3<br><0.0001 vs. 4<br><0.0001 vs. 5<br><0.0001 vs. 6<br><0.0001 vs. 7<br><0.0001 vs. 8	8
2. FANCD2 def	media	Bio-dUTP	900	3	11.56	11.59	<0.0001 vs. 4	8
3. BLM def	media	Bio-dUTP	900	3	9.3	9.18	0.7454 vs 4<br><0.0001 vs. 7	8
4. FANCD2/ BLM def	media	Bio-dUTP	900	3	9.04	9.09	<0.0001 vs. 8	8
5. Wild type	APH	Bio-dUTP	900	3	6.67	6.43	0.7891 vs. 6	8
6. FANCD2 def	APH	Bio-dUTP	900	3	5.99	6.37	<0.0001 vs. 2	8
7. BLM def	APH	Bio-dUTP	900	3	4.08	4.08	<0.0001 vs. 5	8
8. FANCD2/ BLM def	APH	Bio-dUTP	900	3	4.2	4.14	<0.0001 vs. 5	8
9. Wild type	media	Dig-dUTP	900	3	8.12	8.12	0.9457 vs. 10<br><0.0001 vs. 11<br><0.0001 vs. 12<br>0.5386 vs. 13<br><0.0001 vs. 14<br><0.0001 vs. 15<br><0.0001 vs. 16	9
10. FANCD2 def	media	Dig-dUTP	900	3	8.01	8.01	<0.0001 vs. 12	9
11. BLM def	media	Dig-dUTP	900	3	7.11	7.11	0.8463 vs. 15<br>0.3650 vs. 12	9
12. FANCD2/ BLM def	media	Dig-dUTP	900	3	7.4	7.33	<0.0001 vs. 16	9
13. Wild type	APH	Dig-dUTP	900	3	8.02	8.02	<0.0001 vs. 14	9
14. FANCD2 def	APH	Dig-dUTP	900	3	4.05	4.04	<0.0001 vs. 10	9
15. BLM def	APH	Dig-dUTP	900	3	7.18	7.17	<0.0001 vs. 13	9
16. FANCD2/ BLM def	APH	Dig-dUTP	900	3	4.12	4.13	<0.0001 vs. 13	9

Median values are in μm. *Mann-Whitney test.
